# A poisson regression approach for modelling spatial autocorrelation between geographically referenced observations

**DOI:** 10.1186/1471-2288-11-133

**Published:** 2011-10-03

**Authors:** Mohammadreza Mohebbi, Rory Wolfe, Damien Jolley

**Affiliations:** 1 Department of Epidemiology and Preventive Medicine, Faculty of Medicine, Nursing and Health Sciences, Monash University, Melbourne, Australia

**Keywords:** Cancer incidence, Dietary pattern, Disease mapping, Multilevel generalised linear model, Socio-economic status, Spatial analysis

## Abstract

**Background:**

Analytic methods commonly used in epidemiology do not account for spatial correlation between observations. In regression analyses, omission of that autocorrelation can bias parameter estimates and yield incorrect standard error estimates.

**Methods:**

We used age standardised incidence ratios (SIRs) of esophageal cancer (EC) from the Babol cancer registry from 2001 to 2005, and extracted socioeconomic indices from the Statistical Centre of Iran. The following models for SIR were used: (1) Poisson regression with agglomeration-specific nonspatial random effects; (2) Poisson regression with agglomeration-specific spatial random effects. Distance-based and neighbourhood-based autocorrelation structures were used for defining the spatial random effects and a pseudolikelihood approach was applied to estimate model parameters. The Bayesian information criterion (BIC), Akaike's information criterion (AIC) and adjusted pseudo R^2^, were used for model comparison.

**Results:**

A Gaussian semivariogram with an effective range of 225 km best fit spatial autocorrelation in agglomeration-level EC incidence. The Moran's I index was greater than its expected value indicating systematic geographical clustering of EC. The distance-based and neighbourhood-based Poisson regression estimates were generally similar. When residual spatial dependence was modelled, point and interval estimates of covariate effects were different to those obtained from the nonspatial Poisson model.

**Conclusions:**

The spatial pattern evident in the EC SIR and the observation that point estimates and standard errors differed depending on the modelling approach indicate the importance of accounting for residual spatial correlation in analyses of EC incidence in the Caspian region of Iran. Our results also illustrate that spatial smoothing must be applied with care.

## Background

In general, disease counts in areas that are geographically proximate will display residual spatial dependence; 'residual' here acknowledging that dependence of these counts on known risk factors has been taken account of in the analysis model. Spatially correlated observations do not satisfy the independence assumption central to generalized linear model (GLM) theory [1]. However, generalized linear mixed models (GLMMs) can accommodate spatial random effects and provide a flexible means of analysing spatially correlated disease counts [2]. Left unaddressed, residual spatial correlation can bias regression parameter estimates and cause standard errors to be underestimated, leading to confidence intervals that are too narrow and, potentially leading to incorrect inferences regarding exposure-disease associations [3].

The purpose of this paper is to demonstrate for medical statisticians and health researchers how to fit common types of GLMMs with spatially autocorrelated random effects. While Bayesian or frequentist approaches can be used, we limit attention to frequentist approaches here. Most applications of GLMMs use approximate likelihood methods to estimate model parameters. Rather than try to cover a broad array of models (without providing sufficient depth for the reader to understand the logic behind the model), we focus on Poisson regression with three of the most common autocorrelation structures: Poisson regression with agglomeration-specific nonspatial random effects; Poisson regression with distance-based agglomeration-specific spatial random effects; and Poisson regression with neighbourhood-based agglomeration-specific spatial random effects. We aim to compare these three autocorrelation structure approaches for Poisson regression models. In this study these methods are illustrated by investigating the association between the geographic pattern of esophageal cancer, EC, incidence and socio-economic pattern in the Caspian region of Iran.

The structure of this paper is as follows: In the methods section, the EC study's setting and data collection are described and exploratory analysis methods are detailed; then regression models for count data incorporating different assumptions about spatial correlation are presented. In the results section, the application of the different models to the EC data is described, and finally conclusions from these analyses are presented.

## Methods

### Study Population

**Figure 1:**
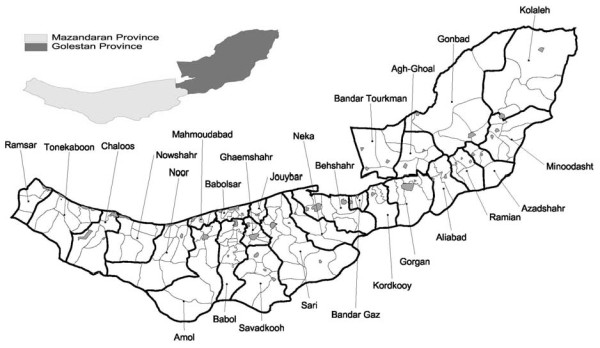
**Geographic boundaries of wards (bold polygons), and cities (gray polygons) and rural agglomerations within wards, in Mazandaran and Golestan provinces**.

Residents of Mazandaran and Golestan provinces constitute the study population (see Figure 1). The estimated midyear population of Mazandaran and Golestan provinces between 2001 and 2005, stratified for age in five-year intervals and place of residence, was obtained from the statistical centre of Iran [4]. These estimates were projections for 2001 to 2005 based on 1995 census data using the 2000 geographic boundaries [5,6]. Geographic coordinates for each agglomeration were also obtained that approximately reflected the geographical centroid of each agglomeration [4].

### Geographic region

Iran has high rates of EC (esophageal cancer) [7,8]. Strong spatial aggregations in esophageal cancer have been identified in the two study provinces with a tendency for high rates in eastern and central wards and low rates in the west [9]. A recent study showed EC was associated with aggregated risk factors related to socio-economic status (SES) including income and urbanisation [10]. The total population of these two provinces is approximately 4.5 million (1.6 million in Golestan province) [4]. The provinces of Iran are subdivided into wards. There are usually a few cities and rural agglomerations in each ward. Rural agglomerations are a collection of a number of villages. Currently, Mazandaran province has 15 wards, 46 cities and 110 agglomerations and Golestan province has 11 wards, 24 cities and 50 agglomerations. Figure 1 shows geographic boundaries of cities and rural agglomerations within wards in Mazandaran and Golestan provinces.

### Data sources

The cases of interest were all EC patients registered between 2001 and 2005 among the study population. The results for both sexes combined are presented in this paper. Data on incident cases of cancer were obtained from the Babol Cancer Registry; issues related to methods, quality and completeness of data collection for this cancer registry are described elsewhere [9,11]. In summary, the major sources of data collection related to cancer in the Babol cancer registry were reports from pathology laboratories, hospitals, and radiology clinics.

For each agglomeration the following socio-economic variables were obtained from the 1995 statistical yearbooks of Mazandaran and Golestan [5,6] and the income and expenses survey in urban and rural areas in 1995 [12,13]: population density (inhabitants per square kilometre), relative level of activity (a synthetic indicator devised by the statistical centre of Iran that is calculated from the number of households, number of telephone lines, number of bank offices, number of commercial licences, electricity consumption, annual construction budget), annual income per family, annual expenditure on food per family, annual expenditure on fruit and vegetables per family, percentage of occupation in the industrial sector, percentage of occupation in the services sector, percentage of occupation in the agricultural sector, percentage of occupation in the construction sector, percentage of male unemployment, and percentage of illiteracy for males and females. In addition to the villages, some agglomerations contain one or more cities; a proportional as-likely basis method was used to calculate socio-economic characteristics of these agglomerations.

### Factor analysis of socio-economic variables

A factor analysis was performed to synthesize the socio-economic variables into a few independent and uncorrelated factors. A Varimax rotation with Kaiser normalisation was used to facilitate interpretation of the factors. The Anderson-Rubin method was used to create factor scores from the factor solution [14]. The factor scores extracted with this method are uncorrelated with a zero average and variance of one. We attached labels to the factors by interpreting coefficients of the items. This process identified three factors: income, urbanisation and literacy. Scores for these factors for each agglomeration were subsequently used in regression models.

### Standardised incidence rates calculation

Adjustment of incidence rates for differences in the age structure of agglomerations was accomplished by age-standardisation. The SIR for an agglomeration was obtained from the ratio of the observed and expected number of cases in that agglomeration. The indirect method of standardisation was used for internal comparisons[15]. Since the population of the region was stable between 2001 and 2005, the 2003 population size was used for computing the incidence rates in age categories of the overall region and the subsequent expected number of cases in each agglomeration. Five-year intervals were used for age categorisation.

### Exploratory spatial data analysis

Two methods were used to measure spatial aggregation of the agglomeration SIRs; Moran's I [16] and semivariograms [17]. Moran's I was adjusted for agglomeration counts by comparing the observed count in a region with its expected count under the constant risk hypothesis [18].

To calculate the empirical semivariogram, we used studentised residuals (residuals that are each divided by their estimated standard error) obtained from a weighted least squares (WLS) regression. This involved a linear regression model of the form

(1) $${\text{Z}}_{\text{i}}=\alpha_{0}+\in_{\text{i}}$$

where α_0 _was an intercept parameter to be estimated,∈_i _was the residual error of the i^th ^agglomeration, and Z_i _was a Box-Cox transformation of the SIRs. The weights used in parameter estimation by WLS were proportional to the population size at risk within each agglomeration. To obtain a succinct statistical description of the spatial correlation three different parametric models (exponential, Gaussian, and spherical) were fitted to the empirical semivariogram, each of which can be described in terms of nugget, sill and range parameters[17]. The model considered most appropriate was that which minimized the residual sum of squares between the theoretical model and the empirical semivariogram [17].

### Analytic methods

Three approaches for modelling with agglomeration-specific random effects were considered: (1) a standard Poisson GLMM with independent agglomeration-specific random effects (nonspatial Poisson GLMM); (2) a discrete autocorrelation structure for agglomeration-specific random effects (neighbourhood-based spatial Poisson GLMM); and (3) a continuous autocorrelation structure for agglomeration-specific random effects (distance-based spatial Poisson GLMM).

For all approaches it was assumed that, conditional on random effects, the number of cancer cases in each of the N agglomerations, Y_1_, . . . , Y_N_, were independent Poisson random variables each with mean μ_i_.

The models contained income, urbanisation and literacy. The aim of the analyses was to identify macro scale SES factors that determine the distribution of EC at the agglomeration level.

(2) $$\begin{array}{c} \log\left(\mu_{\text{i}}\right)=\log\left({\text{E}}_{\text{i}}\right)+{\left(\text{X}\beta \right)}_{\text{i}}\\ {\left(\text{X}\beta \right)}_{\text{i}}=\beta_{0}+{\text{X}}_{\text{SES}}\;\beta_{\text{SES}}\;\;\;\;\;\;\; \end{array}$$

where X_SES _is a design matrix for the three socio-economic factors; β_0 _is an intercept parameter to be estimated, β_SES _is a vector of parameters that describe the socio-economic factor effects on EC and the offset term log(E_i_) is the logarithm of the expected number of cases for that agglomeration (assumed fixed). Since theoretical $\text{SIR}=\frac{\mu_{\text{i}}}{{\text{E}}_{\text{i}}}$, this is a model for observed agglomeration level SIRs and the parameters β_SES _describe association between factor scores and (log) SIR with exp(β_SES_) interpretable as relative risk parameters within each agglomeration.

### Nonspatial Poisson generalized linear mixed model (nonspatial GLMM)

A nonspatial GLMM for the number of cases can be specified as

(3) $$\log\left(\mu_{\text{i}}\right)=\log\left({\text{E}}_{\text{i}}\right)+{\left(\text{X}\beta \right)}_{\text{i}}+{\text{u}}_{\text{i}}$$

where u_i _is the random effect (one for each agglomeration). These are independent random effects, and as per convention, were assumed to be distributed as N(0, $\zeta_{{}_{\text{i}}}^{2}$)[2]. The parameter $\zeta_{{}_{\text{i}}}^{2}$ indicates the variance in the population distribution, and therefore the degree of heterogeneity of agglomerations. These random effects represent the influence of agglomeration i that is not captured by the observed covariates.

### Neighbourhood-based spatial Poisson generalized linear mixed model (neighbourhood-based GLMM)

The neighbourhood-based GLMMs took the following form:

(4) $$\log\left(\mu_{\text{i}}\right)=\log\left({\text{E}}_{\text{i}}\right)+{\left(\text{X}\beta \right)}_{\text{i}}+\Theta_{\text{i}}$$

The vector of random effects Θ_i _was assumed to have conditional autoregressive structure (CAR) [19,20]. A generalization of the CAR called the intrinsic conditional autoregressive structure (ICAR) was used here, where the conditional distribution of the random effects Θ_i _is

(5) $$\Theta_{\text{i}}|\Theta_{\text{j}}\sim \text{N}\left(\bar{\Theta_{\text{i}}},\;\frac{\sigma^{2}}{{\text{n}}_{\text{i}}}\right).$$

where$\bar{\Theta_{\text{i}}}=\sum_{\text{j}\in \delta_{\text{i}}}\frac{\Theta_{\text{j}}}{{\text{n}}_{\text{i}}}$, n_i _is the number of neighbours for agglomeration i, and δ_i _indicates the neighbourhood of agglomeration i [21]. Using the properties of the multivariate normal distribution, Eq. (4) can be specified in a joint formulation, where $\text{Q}=\sum_{\Theta }^{-1}\;\left(1-\text{W}\right)$ is the precision matrix as Θ ~ MVN(0, (σ^-2^Q)^-1^). The diagonal matrix Σ_Θ _has entries $\frac{1}{{\text{n}}_{\text{i}}}$ and W has entries ${\text{W}}_{\text{ij}}=\left\{\begin{array}{c} \frac{1}{{\text{n}}_{\text{i}}}\;\text{if}\;\text{j}\in \delta_{\text{i}}\\ 0\;\text{otherwise} \end{array}\right)$.

In this way, the precision matrix has a rather simple form in ICAR, with the number of neighbours n_i _on the diagonal and off-diagonal entries -1, where i and j are neighbours and zero otherwise.

### Distance-based spatial Poisson generalized linear mixed models (distance-based GLMMs)

Distance-based GLMMs took the following form

(6) $$\log\left(\mu_{\text{i}}\right)=\log\left({\text{E}}_{\text{i}}\right)+{\left(\text{X}\beta \right)}_{\text{i}}+\Phi_{\text{i}}$$

The vector of random effects was assumed to be MVN(0, Σ_Θ_(θ)) with parameters that are jointly referred to as$\Theta \;=\;\left[\begin{array}{c} \tau^{2}\\ \nu^{2}\\ \varphi \end{array}\right]$. If d_ij _denotes the distance between agglomeration centroid i and j, where counts y_i _and y_j _were observed, then

(7) $$\Sigma_{\Phi }\left(\Theta \right)\;=\;\text{I}\tau^{2}\;+\;\text{F}\nu^{2}$$

where the matrix F has elements F_ij _given by $\exp\;\left(\frac{-{\text{d}}_{\text{ij}}}{\varphi }\right)$ for exponential, $\exp\;\left(\frac{-{\text{d}}_{{}_{\text{ij}}}^{\text{2}}}{\varphi^{2}}\right)$ for Gaussian, and $\exp\;\left[-\frac{2}{3}\left(\frac{{\text{d}}_{\text{ij}}}{\varphi }\right)\;-\frac{1}{2}{\left(\frac{{\text{d}}_{\text{ij}}}{\varphi }\right)}^{3}\right]$ for spherical semivariogram models. The unknown parameters in these models, namely, τ^2 ^(the nugget), ν^2 ^(the sill), and φ (the range), can be obtained from a variogram of the data.

### Parameter Estimation

We used a pseudolikelihood approach to estimate unknown parameters in the GLMMs [22]. This approach first assumed that Δ, the vector of variance-covariance parameters, was fixed and used maximum likelihood to estimate β; and then, from the residuals, r, revised the estimate of Δ and iterated until convergence. The following steps were carried out:

1. An initial estimate ${\hat{\beta }}_{0}$ of β was made by assuming no spatial correlation, that is, with Σ as a diagonal matrix.

The deviance residuals r_0 _were calculated using ${\hat{\beta }}_{0}$, and Δ was estimated by using maximum likelihood.

2. A new set of estimates ${\hat{\beta }}_{1}$was found with Δ assumed fixed at the values ${\hat{\Delta }}_{0}$; hence, a new set of deviance residuals r_1 _was calculated.

3. These estimates were used in a new cycle to redraw Δ and thus derive fresh estimates${\hat{\Delta }}_{1}$.

Steps 2 and 3 formed an iterative cycle that continued until there was no further change in the estimates.

### Model comparison

The -2 Log-Likelihood statistic and two commonly used penalized model selection criteria, the Bayesian information criterion (BIC) and Akaike's information criterion (AIC), were used for model comparison. Adjusted pseudo R^2 ^was also used to compare the different models with

$${\text{R}}^{2}=1-\frac{\sum {\text{y}}_{\text{i}}\log\left(\frac{{\text{y}}_{\text{i}}}{{\hat{\lambda }}_{\text{i}}}\right)-\left({\text{y}}_{\text{i}}-{\hat{\lambda }}_{\text{i}}\right)}{\sum {\text{y}}_{\text{i}}\log\left(\frac{{\text{y}}_{\text{i}}}{\bar{\text{y}}}\right)}$$

where y_i _was the observed count and ${\hat{\lambda }}_{\text{i}}$was the model expected count, and adjusted pseudo R^2 ^was given by ${\text{R}}_{{}^{\text{adj}}}^{\text{2}}\;=\;1-\frac{\text{N}-1}{\text{d}\text{.f}.}\;{\left(1\;-\;\text{R}\right)}^{2}$. The adjustment was for the number of degrees of freedom (d.f. = N-no. of model parameters)[23].

### Cartographic display

**Figure 2:**
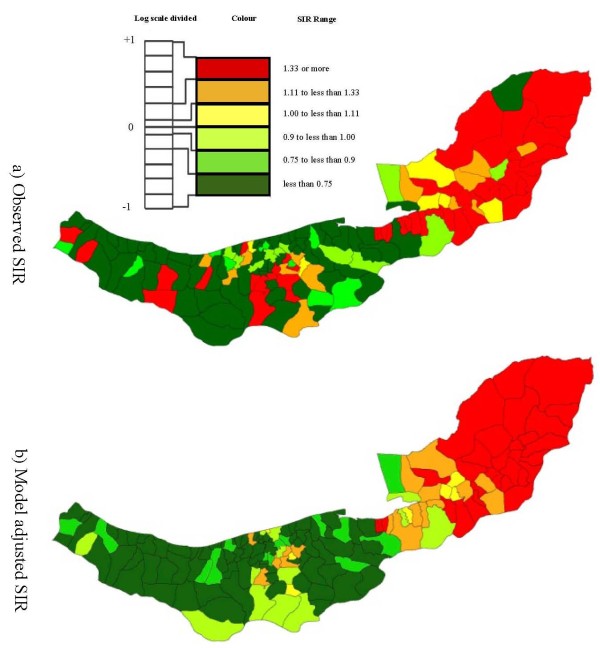
**Observed spatial pattern (a), and neighbourhood-based spatial Poisson generalized linear mixed model adjusted spatial pattern of esophageal cancer SIRs (b)**.

In this study the RR (risk ratio) break points were determined by considering values in the range 0.1 to 10. This corresponds to the range -1 to +1 upon logarithmic transformation. Then this logarithmic scale was divided into 11 equal intervals centred on zero, the break point values were transformed back to the original RR scale, and the five middle intervals were used in the maps. As shown in Figure 2, the middle category was further divided above and below 1. A red-green colour scheme was used for the maps, with shading of red for areas with the highest SIR (> 1.33), followed by orange and yellow for areas with moderately elevated SIR, light and medium green for areas with moderately low SIR, and dark green representing areas with the lowest SIR (< 0.75).

### Software

SIR calculation was done by Microsoft Excel, exploratory spatial analyses were performed using the SAS VARIOGRAM Procedure[24], factor analyses performed using SPSS 17 and the SAS Glimmix procedure was used to carry out the random effect regression models [25,26].

## Results

### Factor analysis

Factor analysis identified three factors with eigenvalues greater than 1. Table 1 shows the correlations between socio-economic items and the extracted factors. The three factors account for 53% of total variance in socio-economic variables and individually the factors account for: income: 25%, urbanisation: 15% and literacy: 13%.

**Table 1:** Socio-economic loadings from factor analysis (Income, Urbanisation and Literacy)*

Rotated Component Matrix			
**Items**	**Components**		
	
	**Income**	**Urbanisation**	**Literacy**

Annual income per family	.846	-	-
Annual expenditure on food per family	.654	.165	-
Annual expenditure on fruit and vegetables per family	.455	.151	-
Population density	-	.285	-
Relative level of activity	.318	.221	.533
% of male unemployment	-.321	-.679	-
% of employment in agriculture	-.213	-.808	-
% of employment in industry	.199	.341	-
% of employment in construction	-.208	-	.470
% of employment in services	.189	.824	-.198
Female illiteracy	-	-	-.642
Male illiteracy	-	-	-.669
* Loadings less than 0.10 in absolute value are not displayed			
			

### Exploratory analysis

A total of 1693 new EC cases were diagnosed in 2001-2005 in Mazandaran and Golestan. The observed Moran index was 0.22 which was greater than its expected value -0.0066, indicating systematic clustering in the region. Consistent with Moran's I, Figure 2(a) showed strong spatial aggregations, with a tendency for high rates in the eastern and central agglomerations and low rates in the west.

**Figure 3:**
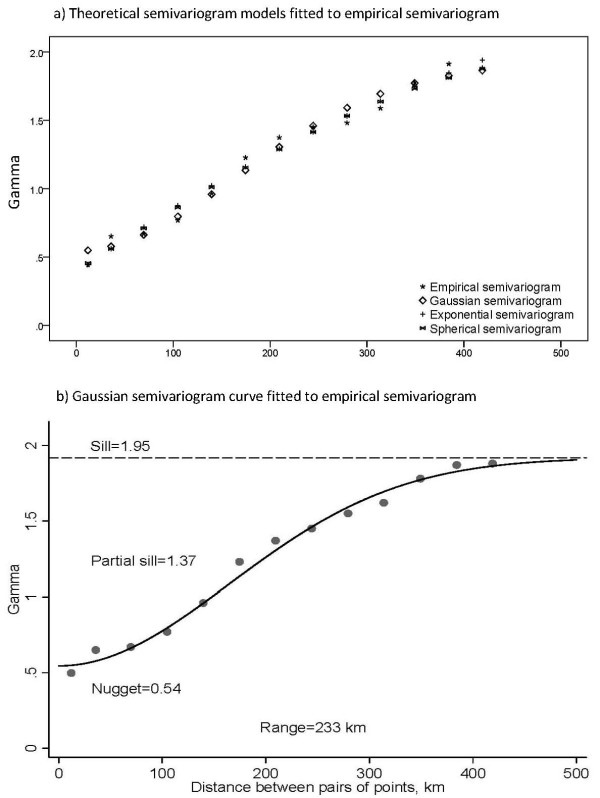
**Empirical semivariogram and theoretical semivariogram values (a), and Gaussian semivariogram fit to the empirical semivariograms points (b)**.

A Gaussian semivariogram best fitted the empirical semivariogram as illustrated in Figure 3(a). The effective range of spatial autocorrelation was 225 km. The nugget/sill ratio was 0.28, indicating a moderate degree of spatial autocorrelation (Figure 3(b)).

### Spatial regression

As illustrated in Figure 3(b), exponential, Gaussian and spherical semivariogram models smoothed out the fluctuations of, and provided a reasonable overall fit to, the empirical semivariogram. Hence we used all three models for Σ_Φ_(θ) and compared the results in Table 2. While the Gaussian model had slightly better fit, the choice of autocorrelation structure had little effect on the results from the distance-based GLMMs. We decided to select the Gaussian distance-based GLMM for further comparisons.

**Table 2:** Comparison of distance-based autocorrelation structures for Poisson GLMM regression using exponential, Gaussian and spherical functions

Model	Parameter			Goodness of fit method		
	
	Nugget	Partial sill	Range	-2Log- Likelihood	AIC	BIC
Exponential distance-based	0.38	3.20	427.7	380.9	390.9	392.9
Gaussian distance-based	0.45	1.21	224.6	373.4	370.5	362.1
Spherical distance-based	0.40	1.65	380.5	381.5	387.5	386.5

**Figure 4:**
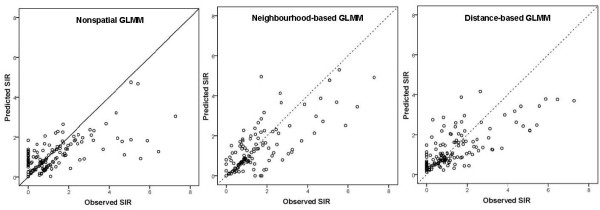
**Scatter plots of observed SIRs (horizontal axis) against model predicted SIRs (vertical axis)**.

Comparison of the overall fit of nonspatial and spatial regression approaches is provided in Table 3. Based on the likelihood ratio, AIC and BIC the neighbourhood-based autocorrelation structure had the best fit to observed EC counts compared to the competing methods. The Pseudo-R^2 ^diagnostic suggested that nearly 25% of the total variation in EC counts could be explained by the three SES factors. This measure also indicated best fit for neighbourhood-based Poisson regression. Figure 4 shows the scatter plots of the observed SIR against the model predicted SIRs for each of the three models. Large observed SIR are smoothed towards 1 in all three approaches, i.e. for those agglomerations model predicted SIRs are closer to 1 and the agglomeration is represented in Figure 4 with a point below the line of equality. This smoothing was most marked in the nonspatial model and least marked in the neighbourhood-based model.

**Table 3:** Comparison of Poisson regression goodness of fit using nonspatial generalized linear mixed model and spatial Poisson generalized linear mixed models with either neighbourhood based or distance based autocorrelation structures

Model	Goodness of fit method			
	
	-2Log- Likelihood	AIC	BIC	Adjusted pseudo R^2 ^
Nonspatial GLMM	413.5	388.5	401.5	18.7%
Neighbourhood-based GLMM	353.0	347.1	357.0	24.7%
Distance-based GLMM	373.4	370.5	362.1	21.2%

Table 4 presents the point and interval estimates for parameters of interest in the nonspatial GLMM, neighbourhood-based GLMM and distance-based GLMM. The two spatial models had RR estimates that were qualitatively similar to the nonspatial GLMM model in finding all three risk factors to be, if anything, protective. However the two spatial GLMMs' RR estimates were considerably further from the null than their nonspatial counterparts, a point returned to below.

**Table 4:** Comparison of Poisson regression results using nonspatial GLMM and spatial GLMMs with neighbourhood based or distance based autocorrelation structure

	Nonspatial GLMM					Neighbourhood-based GLMM					Distance-based GLMM				
**Factor**	**RR**	SE*	**95% CI**		**P-value**	**RR**	SE*	**95% CI**		**P-value**	**RR**	SE*	**95% CI**		**P-value**
												
			**lower**	**upper**				**lower**	**upper**				**lower**	**upper**	

Literacy	1.00	0.11	0.90	1.11	0.99	0.88	0.21	0.58	1.33	0.21	0.93	0.25	0.57	1.52	0.39
Income	0.83	0.22	0.67	1.03	0.20	0.67	0.29	0.50	0.89	0.04	0.74	0.23	0.59	0.93	0.06
Urbanisation	0.78	0.19	0.65	0.94	0.08	0.55	0.35	0.39	0.77	0.05	0.70	0.22	0.56	0.87	0.05

* Standard error of RR

In general, 95% CIs for relative risks in the two spatial GLMMs were wider than the corresponding intervals in the nonspatial GLMM, reflecting the reasonably strong inter-agglomeration correlation being taken into account by the spatial model approaches and corresponding effective reduction in sample size in comparison with the nonspatial model that assumes independence of these agglomerations.

As mentioned above, the neighbourhood-based GLMM and distance-based GLMM point estimates were systematically smaller compared with the nonspatial GLMM. This attenuation of effect away from the null was especially marked for the income and urbanisation factors.

The neighbourhood-based GLMM and distance-based GLMM RR estimates had comparable conclusions based on inspection of p-values but all three associations were more strongly protective in the neighbourhood-based model. The results for the neighbourhood-based autocorrelation structure indicated that an increase in the score of the income or urbanisation factor was significantly associated with decreasing EC SIR. Model smoothed SIR maps after adjustment for the three factors in Table 3 in a neighbourhood-based GLMM are illustrated in Figure 2(b).

## Discussion

In this ecologic study we observed significant associations between agglomeration-specific EC SIR and SES. The geographic pattern of cancer SIR was consistently stronger among eastern regions and among rural agglomerations. However this conclusion depended on the choice of model.

Multiple forms of residual correlation were potentially present in the cancer SIR data; we considered models addressing 2 types of geographical correlation. A semivariogram plot confirmed the presence of substantial distance-based residual spatial correlation in the EC SIR; agglomerations greater than 225 km apart behaved essentially as independent observations in EC, but at lesser distances, SIR were increasingly correlated as the distance between agglomerations centroids diminished. The global Moran's I showed the importance of neighbourhood-based residual correlation in the data. CAR spatial smoothing works by making neighbours more alike than non-neighbours, and careful consideration must be given to the way in which neighbours are defined. By defining neighbours based on sharing a border, artificial similarities between some agglomerations may have been induced and would have had the effect of producing some attenuation of fixed-effect estimates. However there was little evidence of this in Figure 4.

The studentised residuals should approximately have a zero mean and a constant variance (1), and their distribution should be roughly Gaussian. We found little deviation from these assumptions in the residual analysis of the transformed SIR data (Z_i_'s). Thus, the studentised residuals should approximately satisfy the assumption of intrinsic stationary. As discussed by Cressie [17], semivariogram estimators based on the residuals are biased and in this case the covariance of the error could depend on the agglomerations' population sizes. We weighted the semivariogram parameter estimation by agglomeration population size to minimise this bias. When the aim of semivariogram estimation is perfection such as in the Poisson kriging method, Poisson semivariogram estimation taking into account the size of the administrative units can be used [27].

Useful information for model selection can be obtained from using AIC and BIC together, particularly from trying as far as possible to find models favoured by both criteria. Quantitatively, the BIC puts more penalty on the log-likelihood function and the BIC favours more parsimonious models [28]. In the present study the neighbourhood-based Poisson regression was favoured by AIC, BIC and adjusted pseudo R^2 ^indices, although the distance-based Poisson model was closer to the neighbourhood-based GLMM results than was the nonspatial Poisson regression.

Ignoring spatial autocorrelation can result in explanatory variables apparently being associated with incidence as a result of overstatement of the degrees of freedom in the data and consequent underestimation of standard errors. In our analysis, the standard errors of the regression coefficients were smaller by as much as 35 percent in the model that ignored spatial effects compared with the models that adjusted for spatial effects. As a result, accounting for spatial autocorrelation increased the width of confidence intervals for point estimates. We also observed modest attenuation of SES factor associations with EC in the direction of the null, i.e. a risk ratio of 1, in the nonspatial model. In general it is difficult to predict the direction and nature of this attenuation when a spatial random effect is added to model [29]. Results of simulation studies suggest that the importance of accounting for spatial autocorrelation will depend on the similarity in the spatial variation of the agglomeration-level exposure with the extra-Poisson variation in the outcome, as well as the strength of that correlation. If the agglomeration-level exposure varies on a larger scale over space relative to the spatial structure of the residuals, then ignoring the spatial autocorrelation may lead to underestimation of the exposure effect. If the spatial structures of exposure and disease incidence are similar, then the exposure effect may be confounded by the inclusion of spatial random effects [30-32].

It is noteworthy that the neighbourhood-based GLMM is optimal if all agglomerations are of similar size and arranged in a regular pattern and the distance-based GLMM is optimal if all inhabitants of each agglomeration live at their agglomeration's centroid and the measured rate thus refers to this specific location. There have been attempts to model Poisson kriging accounting for size and shape of administrative units as well as population density [33,34].

Bayesian methods have been widely used for the analysis of spatially correlated count data using software such as WinBUGS and MLwiN. WinBUGS was developed purely for Bayesian analysis, while MLwiN also allows likelihood approaches to GLMMs. Bayesian models have to specify prior distributions for all parameters and require computationally intensive MCMC sampling. Likelihood and Bayesian approaches for spatial Poisson regression have been compared for several case studies with similar results found [35].

Valid inference in spatial regression requires acknowledgment of residual spatial dependence, since regression coefficients of interest will often be sensitive to the form of the dependence assumed, as was observed in this study. The exploratory data analysis showed significant geographic autocorrelation suggesting that the nonspatial GLMM, which ignored this type of correlation, was inappropriate. In the nonspatial GLMM presented here, the agglomeration-specific random effects, assumed to be independent, introduced extra-Poisson variation in EC counts but ignored between-agglomeration spatial correlation. In contrast, the spatial GLMMs explicitly defined spatially-structured random effects, accounting for between-agglomeration spatial correlation but not addressing extra-Poisson variation, which would have required a second set of agglomeration-specific random effects. While we have illustrated the use of SAS, one widely available statistical software package, implementations of these methods also exist in R and WinBugs [25,26].

## Conclusions

Our results illustrate the importance of accounting for residual spatial correlation in analyses of ecologic studies of disease incidence.

GLMMs are accessible, flexible methods that enable epidemiologists to account for residual spatial correlation. With careful definition of correlation structure and careful application of spatial smoothing, spatial GLMMs are a valuable tool to account explicitly for the effects of residual spatial correlation during the regression modelling process.

## List of abbreviations used

AIC: Akaike's information criterion; BIC: Bayesian information criterion; CAR: Conditional autoregressive; Distance-based GLMM: Distance based spatial Poisson generalized linear mixed model; EC: Esophageal cancer; GLM: Generalized linear model; GLMM: Generalized linear mixed model; ICAR: Intrinsic conditional autoregressive; Neighbourhood-based GLMM: Neighbourhood based spatial Poisson generalized linear mixed model; Nonspatial GLMM: Nonspatial Poisson generalized linear mixed model; RR: Risk ratio; SES: Socio-economic status; SIR: Standardised incidence ratio; WLS:Weighted least squares

## Competing interests

The authors declare that they have no competing interests.

## Authors' contributions

MM was responsible for the data collection process and issues related to data quality. MM performed the statistical analysis. MM and RW wrote the first draft of the manuscript to which all authors subsequently contributed. All authors read and revised the manuscript for important intellectual content and approved the final manuscript.

## Pre-publication history

The pre-publication history for this paper can be accessed here:

http://www.biomedcentral.com/1471-2288/11/133/prepub

## References

[B1] McCullagh P Nelder JA Generalized Linear Models 1989 London: Chapman and Hall

[B2] Breslow NE Clayton DG Approximate inference in generalized linear mixed models J Am Stat Assoc 1993 88 9 25 10.2307/2290687

[B3] Waller L Gotway C Applied Spatial Statistics for Public Health Data 2004 New York: Wiley

[B4] Iran statistical yearbook 2000 Tehran: Statistical Center of Iran

[B5] Reconstruction and estimation of Golestan province population according to 2000 geographic boundaries 2003 Tehran: Statistical Center of Iran

[B6] Reconstruction and estimation of Mazandaran province population according to 2000 geographic boundaries 2003 Tehran: Statistical Center of Iran

[B7] Mahboubi E Kmet J Cook P Day N Ghadirian P Salmasizadeh S Oesophageal cancer studies in the Caspian Littoral of Iran:the caspian cancer registry Br J Cancer 1973 28 197 214 10.1038/bjc.1973.138 4743904 PMC2008981

[B8] Saidi F Sepehr A Fahimi S Farahvash MJ Salehian P Esmailzadeh A Keshoofy M Pirmoazen N Yazdanbod M Roshan MK Oesophageal cancer among the Turkomans of northeast Iran Br J Cancer 2000 83 1249 1254 10.1054/bjoc.2000.1414 11027442 PMC2363591

[B9] Mohebbi M Mahmoodi M Wolfe R Nourijelyani K Mohammad K Zeraati H Fotouhi A Geographical spread of gastrointestinal tract cancer incidence in the Caspian Sea region of Iran: spatial analysis of cancer registry data BMC Cancer 2008 8 137 10.1186/1471-2407-8-137 18479519 PMC2397428

[B10] Mohebbi M Wolfe R Jolley D Forbes A Mahmoodi M Burton R The spatial distribution of esophageal and gastric cancer in Caspian region of Iran: an ecological analysis of diet and socio-economic influences International Journal of Health Geographics 2011 10 13 10.1186/1476-072X-10-13 21324144 PMC3050677

[B11] Mohebbi M Nourijelyani K Mahmoudi M Mohammad K Zeraati H Fotouhi A Moghadaszadeh B Time of occurrence and age distribution of digestive tract cancers in northern Iran Iranian J Publ Health 2008 37 8 19

[B12] Income and expenses survey in rural families in 1995 1996 Tehran: Statistical Center of Iran

[B13] Income and expenses survey in urban families in 1995 1996 Tehran: Statistical Center of Iran

[B14] Anderson T (Ed.) An introduction to multivariate statistical analysis 1984 New York: John Wiley & Sons

[B15] Esteve J Benhamou E Raymond L Descriptive Epidemiology 1994 Iv Lyon: IARC Scientific Publication 7698823

[B16] Moran P Notes on continuous stochastic phenomena Biometrika 1950 37 17 23 15420245

[B17] Cressie N Statistics for Spatial Data, rev. edn 1993 New York: Wiley

[B18] Walter SD The analysis of regional patterns in health data: I. Distributional considerations Am J Epidemiol 1992 136 730 741 10.1093/oxfordjournals.aje.a116552 1442739

[B19] Langford IH Leyland AH Rasbash J Goldstein H Multilevel modelling of the geographical distributions of diseases J R Stat Soc Ser C Appl Stat 1999 48 253 268 10.1111/1467-9876.00153 12294883

[B20] Clayton D Kaldor J Empirical Bayes estimates of age-standardized relative risks for use in disease mapping Biometrics 1987 43 671 681 10.2307/2532003 3663823

[B21] Besag J York J Mollié A Bayesian image restoration with two applications in spatial statistics Ann Inst Stat Math 1991 43 1-20

[B22] Wolfinger R O'Connell M Generalized linear mixed models a pseudo-likelihood approach Journal of Statistical Computation and Simulation 1993 48 233 243 10.1080/00949659308811554

[B23] Cameron A Windmeijer F R^2 ^measures for count data regression models with applications to health-care utilization Journal of Business and Economic Statistics 1996 14 209 220 10.2307/1392433

[B24] SAS/STAT 9.2 User's Guide, Chapter 95: The VARIOGRAM Procedure 2008 SAS Publishing

[B25] Littell R Milliken G Stroup W Wolfinger R SAS system for mixed models; Chapter 11: Spatial Variability 2006 2 Cary, NC: SAS Institute, Inc21827979

[B26] Rasmussen S Modelling of discrete spatial variation in epidemiology with SAS using GLIMMIX Computer Methods and Programs in Biomedicine 2004 76 83 89 10.1016/j.cmpb.2004.03.003 15313544

[B27] Monestiez P Dubroca L Bonnin E Durbec JP Guinet C Geostatistical modelling of spatial distribution of balaenoptera physalus in the Northwestern Mediterranean sea from sparse count data and heterogeneous observation efforts Ecological Modelling 2006 193 615 628 10.1016/j.ecolmodel.2005.08.042

[B28] Kuha J AIC and BIC comparisons of assumptions and performance Sociological Methods & Research 2004 33 188 22921949579

[B29] Best NG Arnold RA Thomas A Waller LA Conlon EM Bernardo JM, Berger JO, Dawid AP, Smith AFM Bayesian models for spatially correlated disease and exposure data Bayesian statistics 6, eds 1999 Oxford: Clarendon Press 131 156

[B30] Clayton D Bernardinelli L Cuzick J, Elliott P Bayesian methods for mapping disease risks Methods for Small area studies in geographical and environmental epidemiology 1992 Oxford: Oxford University Press 205 220

[B31] Guthrie KA Sheppard L Wakefield J A hierarchical aggregate data model with spatially correlated disease rates Biometrics 2002 58 898 905 10.1111/j.0006-341X.2002.00898.x 12495144

[B32] Wakefield J Salway R A statistical framework for ecological and aggregate studies Journal of the Royal Statistical Society Series A 2001 164 119 137 10.1111/1467-985X.00191

[B33] Goovaerts P Geostatistical analysis of disease data: accounting for spatial support and population density in the isopleth mapping of cancer mortality risk using area-to-point Poisson kriging International Journal of Health Geographics 2006 5 52 10.1186/1476-072X-5-52 17137504 PMC1697809

[B34] Goovaerts p Kriging and semivariogram deconvolution in the presence of irregular geographical units Mathematical Geology 2008 40 101 128 18725997 PMC2518693

[B35] Jang M Lee Y Lawson A Browne W A comparison of the hierarchical likelihood and Bayesian approaches to spatial epidemiological modelling Environmetrics 2007 18 809 821 10.1002/env.877

